# Absolute Quantitative Metagenomic Analysis Provides More Accurate Insights for the Anti-Colitis Effect of Berberine via Modulation of Gut Microbiota

**DOI:** 10.3390/biom15030400

**Published:** 2025-03-11

**Authors:** Jiaguo Zhan, Jiale Cheng, Wenhui Chang, Yuying Su, Xixin Yue, Chongming Wu

**Affiliations:** 1 School of Chinese Materia Medica, Tianjin University of Traditional Chinese Medicine, Tianjin 301617, China; zhanjiaguo8012@163.com (J.Z.); 15565340084@163.com (J.C.); 15632041005@163.com (W.C.); 15092305112@163.com (Y.S.); 16629236959@163.com (X.Y.); 2 State Key Laboratory of Chinese Medicine Modernization, Tianjin 301617, China; 3 Tianjin Key Laboratory of Therapeutic Substance of Traditional Chinese Medicine, Tianjin 301617, China

**Keywords:** gut microbiota, absolute quantitative metagenomic analysis, berberine, meta-analysis

## Abstract

Current gut microbiota studies often rely on relative quantitative sequencing. However, under certain circumstances, while the relative quantitative abundance of these bacteria may remain stable, the absolute quantities of specific bacteria can vary considerably. Since the function of bacteria is directly linked to their total numbers, absolute quantification is crucial. This study aims to identify the optimal method for microbiome analysis by comparing relative and absolute quantitative sequencing. Using ulcerative colitis, which is closely associated with gut microbiota, as a disease model and berberine (which affects microbiota) versus sodium butyrate (which does not) as drugs, relative and absolute quantitative methods were used to evaluate the varying effects of the different drugs on the regulation of gut microbiota in UC-affected animals. The regulatory effects of BBR on gut microbiota were further synthesized as identified in earlier studies using an individual-based meta-analysis, and we compared these findings with our absolute sequencing results. The results from absolute sequencing were more consistent with the actual microbial community, suggesting that relative abundance measurements might not accurately reflect the true abundance of microbial species. Moreover, meta-analysis results were only partially consistent with absolute quantitative sequencing and sometimes directly opposed, suggesting that relative quantitative sequencing analyses are prone to misinterpretation and incorrect correlation of results. This study underscores the importance of absolute quantitative analysis in accurately representing the true microbial counts in a sample and evaluating the modulatory effects of drugs on the microbiome, which plays a vital role in the study of the microbiome.

## 1. Introduction

The gut microbiota significantly influences the onset and progression of various diseases, as well as the effectiveness of treatments [1,2]. However, many studies in this field primarily rely on quantitative methods to analyze shifts in bacterial populations. It is important to note that relative quantification can only provide a limited understanding of the host’s health status. In some cases, the physiological and ecological significance of specific microbial taxa may be overshadowed by their relative abundance, or relative abundance may be the opposite of absolute abundance [3]. A common approach to relative quantification is to calculate relative abundance by summing each sample to 1, resulting in so-called “compositional data”. The inherent problems with “compositional data” obtained with relative quantification have long been recognized, and they are known to lead to spurious correlations between variables. Therefore, the use of relative quantitative analysis of drugs that modulate microbiota may lead to erroneous conclusions or the loss of key bacterial genera [4,5]. Increasing evidence suggests that assessing the total number and concentration of gut microbes (or “absolute abundance”) offers more detailed insights than relative abundance and can help refine conclusions based on relative abundance data [6].

Ulcerative colitis (UC) is a chronic inflammatory bowel disease characterized by an inflammatory imbalance, intestinal epithelial mucosal damage, and intestinal dysbiosis [7]. Sodium butyrate and berberine are recognized for their efficacy in mitigating ulcerative colitis [8]. Mechanistic studies have demonstrated that both compounds ameliorate experimental ulcerative colitis through enhancement of the intestinal barrier, reduction of mesenteric neuronal deficits, and inhibition of inflammation and oxidative stress [9,10,11]. Meanwhile, sodium butyrate (SB) and berberine (BBR) can up-regulate the relative abundance of beneficial bacteria such as *Lactobacillus*, *Roseburia*, *Bacteroides*, and *Akkermansia* and decrease the relative abundance of harmful genera such as *Alloprevotella*, *Eisenbergiella*, and others [12,13]. Berberine is recognized for its antimicrobial properties, while sodium butyrate effectively supports the proliferation of beneficial gastrointestinal bacteria. Consequently, comparing the impacts of berberine and sodium butyrate on gut microbiota proves valuable in discerning differences between relative and absolute quantification methods.

This study aimed to elucidate the differences between relative quantitative and absolute quantitative sequencing by comparing the regulatory effects of SB and BBR on gut microbiota in DSS-induced colitis. Firstly, the ameliorative effects of SB and BBR on DSS-induced colitis were reaffirmed. Subsequently, the differential regulatory effects of the two compounds on gut microbiota were evaluated using both relative and absolute quantitative sequencing methods. Finally, an individual-based meta-analysis was conducted utilizing existing databases to identify the key microbial bacteria regulated by berberine, and we compared our sequencing results with those obtained from the meta-analysis. This study demonstrates that, in comparison to relative quantification, absolute quantitative sequencing can more accurately capture the regulatory effects of drugs on gut microbiota and elucidate the mechanisms of drug action. This finding holds considerable significance for the study of intestinal microorganisms and the development of pharmaceuticals.

## 2. Materials and Methods

### 2.1. Meta-Analysis of BBR-Regulated Gut Microbiota

To systematically obtain data on intestinal flora regulated by berberine, we utilized the PubMed database, employing the keywords “berberine” and “gut microbiota”. The method is consistent with that previously described [14]; the flowchart is shown in Figure S1. Original research articles published in English that contained original data on gut microbiota were selected from Pubmed databases. Following this, we retrieved the FASTQ files using a Linux-based system. Subsequently, quality checks, noise reduction, and species annotation were conducted on the individual datasets to derive operational taxonomic unit (OTU) tables for comprehensive analysis.

### 2.2. Animal Research

Male Kunming mice, acquired from StemBioSys Biotechnology Co., Ltd. (Beijing, China), were hosted in a specific pathogen-free (SPF) environment at Tianjin University of Traditional Chinese Medicine, with a temperature of 24 ± 1 °C, a relative humidity of 50–70%, and a 12 h light/dark cycle. All experimental procedures adhered to the ethical guidelines stipulated by the Academic Committee on Animal Experiment Ethics at Tianjin University of Traditional Chinese Medicine (ethics committee approval number: TCM-LAEC2023234z1619). Forty-eight SPF grade Kunming mice were randomly divided into four groups as follows (*n* = 12/group): Control group, Model group (3% DSS), Berberine group (BBR, 0.2 g/mL), Sodium Butyrate group (SB, 2.85 g/L).

In the first week (5 days of administration in advance), the BBR and SB group were administered 10 g/0.1 mL of the corresponding drugs based on their body weight; all groups were weighed daily and provided with sterile water and a normal diet throughout the period. Starting from the second week, 3% DSS solution was administered in the drinking water to the model group and drug-treated groups to induce ulcerative colitis. The modeling phase was expected to last between 7 to 10 days. The weight of the mice in each group was monitored and recorded starting from the end of the administration period, while the shape of the mice’s feces and hematochezia were assessed.

### 2.3. Calculation of Disease Activity Index (DAI)

The calculation method of the DAI index is consistent with a previous study [14], in which a combination of body weight loss, stool consistency, and the presence of bloody feces were taken into account. The calculation method of DAI is shown in Table 1. DAI = (body weight loss score + stool consistency score + hematochezia score)/3.

### 2.4. Histological Examination

As mentioned earlier [14], colon specimens fixed in 4% paraformaldehyde (Solarbio, Beijing, China) were processed for histopathological evaluation. Following dehydration through an ethanol concentration gradient, tissues underwent paraffin embedding and microtome sectioning. The prepared sections received standard hematoxylin-eosin (H&E) staining protocol prior to microscopic examination. Histopathological scoring (0–4 scale) was implemented according to lesion characteristics: 0 = normal architecture; 1 = mild; 2 = moderate; 3 = severe; 4 = extensive tissue damage, with grading criteria based on pathological severity assessment.

### 2.5. ELISA Assay

Colon tissues from each mouse were homogenized in PBS with 1% PMSF (Solarbio, Beijing, China) using a cryogenic tissue homogenizer. The homogenates were centrifuged at 12,000 rpm for 15 min under 4 °C and the supernatants were collected. Similarly, blood samples underwent centrifugation at 3000 rpm for 15 min at 4 °C to acquire serum. The inflammatory cytokines were then quantified utilizing ELISA kits (Jiangsu Meimian Industrial Co., Ltd., Jiangsu, China) according to the manufacturer’s guidelines.

### 2.6. Full Length 16 S rRNA Gene Sequencing

Fecal DNA extraction was performed following the protocol outlined in previous studies. The primers 27 F 5′-AGRGTTYGATYMTGGCTCAG-3′ and 1492 R 5′-RGYTACCTTGTTACGACTT-3′ were used to amplify the V1-V9 region of the 16S rRNA gene. Amplicons were extracted from 2% agarose gels, and SMRTbell libraries were prepared through blunt-end ligation according to the manufacturer’s instructions (Pacific Biosciences, Menlo Park, CA, USA). The SMRTbell library was then sequenced using individual PacBio Sequel II cells. Amplicon sequencing was carried out by Genesky Biotechnologies Inc. (Shanghai, China). The resulting FASTA sequences underwent quality control and alignment, and an OTU table was created based on a 97% similarity threshold. Further analyses of relative bacterial abundance, as well as alpha and beta diversity, were conducted using R software (version 4.2.3).

### 2.7. Statistical Analysis

The pharmacological results were analyzed by one-way ANOVA using GraphPad Prism version 8 (GraphPad, San Diego, CA, USA) and presented as mean ± standard error of mean (SEM). The microbial data were analyzed by the Wilcoxon rank sum test using the ggpubr package in R software (version 4.2.2). Results were deemed statistically significant when the *p* value was under 0.05.

### 2.8. AI Usage Statement

During the writing process, AI tools were utilized for language refinement and formatting assistance. Specifically, ChatGPT-4 (OpenAI, San Francisco, CA, USA) was employed to polish sentence structures and ensure grammatical accuracy. All intellectual contributions, including data interpretation and conclusions, were independently formulated by the authors.

## 3. Result

### 3.1. BBR and SB Administration Ameliorated the Symptoms of DSS-Induced Colitis in Mice

In order to study whether supplementing berberine and sodium butyrate exerts a protective effect on UC, a DSS mouse model was induced in 6–8 week-old Kunming mice, and berberine and sodium butyrate were administered orally; one week after the administration, 3% DSS was added to the drinking water and the model was established for 9 days. The control group received a regular chewing diet and normal drinking water.

**Figure 1:**
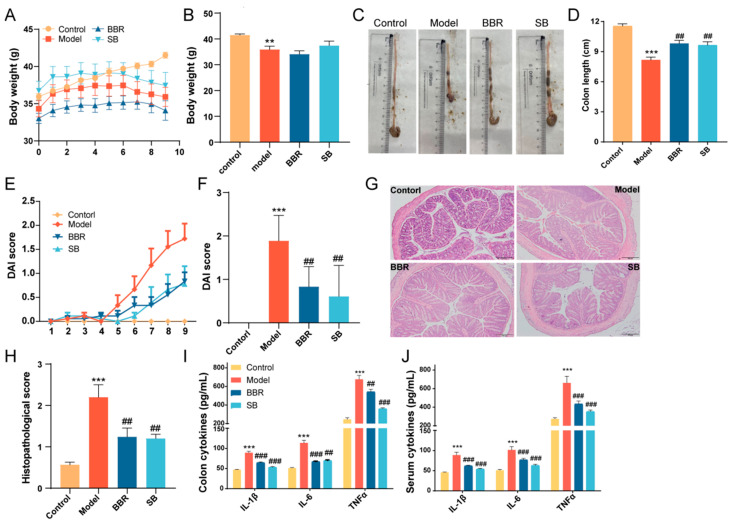
BBR and SB relieves UC in DSS-induced mice. (**A**) Body weight curve. (**B**) Body weight on 9th day. (**C**,**D**) Colon length. (**E**) DAI score curve. (**F**) DAI score on 9th day. (**G**) H&E staining of colon tissue. (**H**) Histological scores of colon among different groups. (**I**) Concentrations of serum pro-inflammatory cytokines. (**J**) Concentrations of colon pro-inflammatory cytokines. *n* = 6 for each group. ** *p* < 0.01, *** *p* < 0.001 vs. Con, ## *p* < 0.01, ### *p* < 0.001 vs. Model.

The weight of mice in the control group continued to increase, as seen in Figure 1A. However, after 6 days of administration of 3% DSS drinking water, the mice experienced a significant weight loss, along with severe diarrhea and bloody stools. Administration of BBR and SB provided relief for some of these symptoms, as shown in Figure 1B. Visual observation revealed that the colon in the DSS group became severely congested and dark in color. In contrast, after BBR and SB administration, the colon appearance of mice with UC returned to normal, accompanied by a reduction in the DAI score (Figure 1C–F). Histological images demonstrated that the colon base in the DSS group became thinner, with broken epithelial cells, reduced crypts and goblet cells, and a compromised colon barrier; treatment with BBR and SB significantly alleviated these symptoms and reduced the colon histopathology score (Figure 1G,H). Given that intestinal and systemic inflammation are common in ulcerative colitis patients, we also investigated the effects of BBR and SB on both colonic and systemic inflammation in the DSS model mice. As shown in Figure 1I,J, administration of BBR and SB significantly inhibited the levels of colon and serum inflammatory factors TNF-α, IL-6, and IL-1β. These findings are consistent with previous experimental results, indicating that both BBR and SB are effective in improving experimental colitis.

### 3.2. Relative Quantitative Sequencing-Based Observation of the Effects of SB and BBR on the Gut Microbiota of DSS Mice

**Figure 2:**
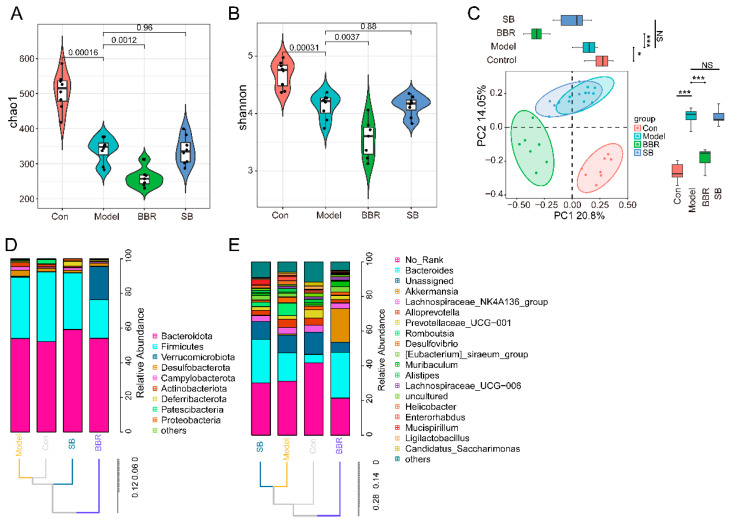
Relative quantitative analysis of the diversity and overall structure of gut microbiota after BBR and SB treatment. (**A**) Chao1 index of relative quantitative analysis. (**B**) Shannon index of relative quantitative analysis. (**C**) Principal coordinates analysis (PCoA) of relative quantitative analysis. (**D**,**E**) The taxonomic profiles of gut microbiota at the phylum (**D**) and genus (**E**) levels of relative quantitative analysis. * *p* < 0.05, *** *p* < 0.001 vs. Model, NS indicates no significant difference.

Chao1 and Shannon indices were utilized to assess community richness and diversity, respectively. There was a significant reduction in species richness and diversity in the community of DSS mice. Compared to the model group, both community diversity and richness were notably decreased after BBR treatment, while alpha diversity remained relatively unchanged after SB treatment (Figure 2A,B). PCOA analysis, based on Bray–Curtis distance, was employed as a measure of beta diversity. The PCOA findings indicated substantial differences in the overall structure of the intestinal flora between the model and control group. Post BBR treatment, the intestinal flora structure diverged significantly from both the normal and model groups. Conversely, the overall intestinal flora structure displayed no significant changes following SB supplementation (Figure 2C). Microbiota composition at the phylum and genus levels was determined using ASV abundance. The primary phyla observed in the four groups were Bacteroidota, Firmicutes, Verrucomicrobiota, and Desulfobacterota. The control, model, and SB groups exhibited similar phylum compositions, while the BBR group showed a decrease in the Firmicutes–Bacteroidota ratio and a significant increase in Verrucomicrobiota abundance. At the genus level, notable differences in abundance were observed among various bacterial genera such as *Bacteroides*, *Akkermansia*, and *Romboutsia* (Figure 2D,E).

**Figure 3:**
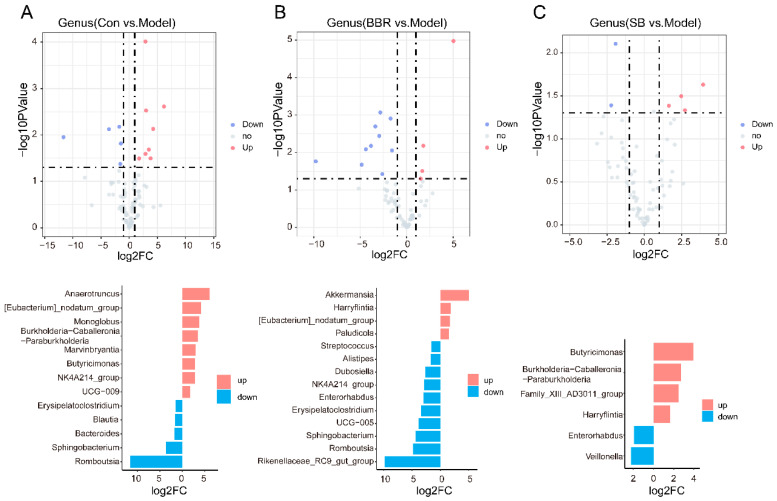
BBR and SB alter core species in UC mice under relative quantification. (**A**) Up-regulated and down-regulated species in DSS groups compared to control group in relative quantitative analysis. (**B**) Volcano map and two-way bar chart showing the core species altered by BBR in relative quantitative analysis. (**C**) Volcano map and two-way bar chart showing the core species altered by SB in relative quantitative analysis.

Volcano plots were utilized to compare bacterial genera with notable changes in abundance between different groups. The abundance of *Romboutsia*, *Sphingobacterium*, *Bacteroides*, *Blautia*, and *Erysipelatoclostridium* were significant increased, while *UCG-009*, *NK4A214_group*, *Butyricimonas*, *Marvinbryantia*, *Burkholderia-Caballeronia-Paraburkholderia*, *Monoglobus*, and *Anaerotruncus* were significant decreased in DSS mice (Figure 3A). After treatment with BBR, there was a significant down-regulation in the abundance of *Rikenellaceae_RC9_gut_group*, *UCG-005*, *Enterorhabdus*, *NK4A214_group*, *Dubosiella*, *Alistipes*, and *Streptococcus*, with *Akkermansia*, *Paludicola*, and *Harryflintia* showing an increase (Figure 3B). SB treatment led to a significant increase in the abundance reduction of *Burkholderia-Caballeronia-Paraburkholderia*, and *Butyricimonas* caused by DSS and also increased the abundance of *Harryflintia* and *Family_XIII_AD3011_group*, while decreasing the abundance of *Veillonella* and *Enterorhabdus* (Figure 3C).

### 3.3. Absolute Quantitative Sequencing-Based Observation of the Effects of SB and BBR on the Gut Microbiota of DSS Mice

**Figure 4:**
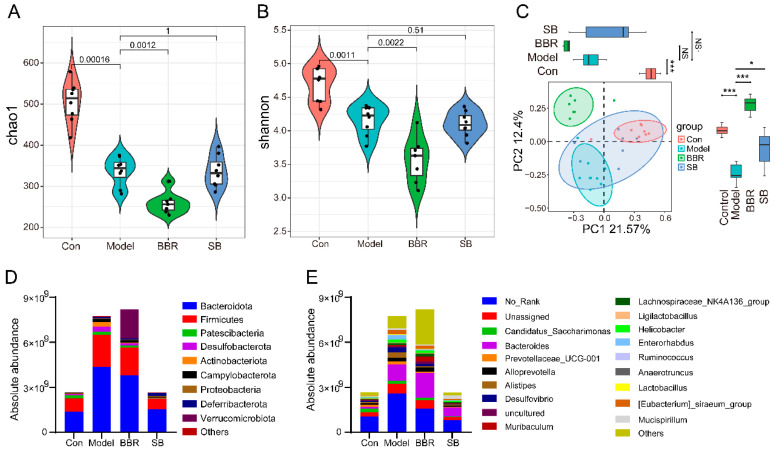
Absolute quantitative analysis of the diversity and overall structure of gut microbiota after BBR and SB treatment. (**A**) Chao1 index of absolute quantitative analysis. (**B**) Shannon index of absolute quantitative analysis. (**C**) Principal coordinates analysis (PCoA) of absolute quantitative analysis. (**D**,**E**) The taxonomic profiles of gut microbiota at the phylum (**D**) and genus (**E**) levels of absolute quantitative analysis. * *p* < 0.05, *** *p* < 0.001 vs. Model, NS indicates no significant difference.

The community richness and diversity were evaluated by using Chao1 and Shannon indices. Consistent with the results of the relative quantitative, there was a significant reduction in species richness and diversity in DSS mice; BBR treatment led to a significant decrease in community richness and diversity compared to the model group. However, no significant change in alpha diversity was observed after SB supplementation (Figure 4A,B). Beta diversity was evaluated through PCoA using Bray–Curtis distance, revealing significant differences in the overall structure of intestinal flora between the model group and control group. Furthermore, the overall structure of intestinal flora post-BBR supplementation differed significantly from both the normal and model groups, while the structure post-SB supplementation shifted towards that of the normal group (Figure 4C).

The main phyla in all groups were Bacteroidota, Firmicutes, Patescibacteria, and Desulfobacterota, but their absolute abundance was not the same in several groups. Among them, the total number of species in the model group and the BBR group far exceeded that of the control group; this may be due to increased cecal inflammation, which slows down the peristalsis and emptying of the cecum, further leading to the accumulation and growth of bacteria, while BBR promotes the proliferation of verrucomicrobiota phylum. Interestingly, the total number of species and the compositional structure of the SB group were close to those of the control group (Figure 4D). At the genus level, the total number of species in the BBR group far exceeded that of the control group, especially the number of *Bacteroides*, *Desulfovibrio*, and *Helicobacter*; the total number of species in the SB group was close to that of the control group, but the number of some genera showed a large difference between the two groups, e.g., *Bacteroides*, *Mucispirillum*, and so on (Figure 4E).

**Figure 5:**
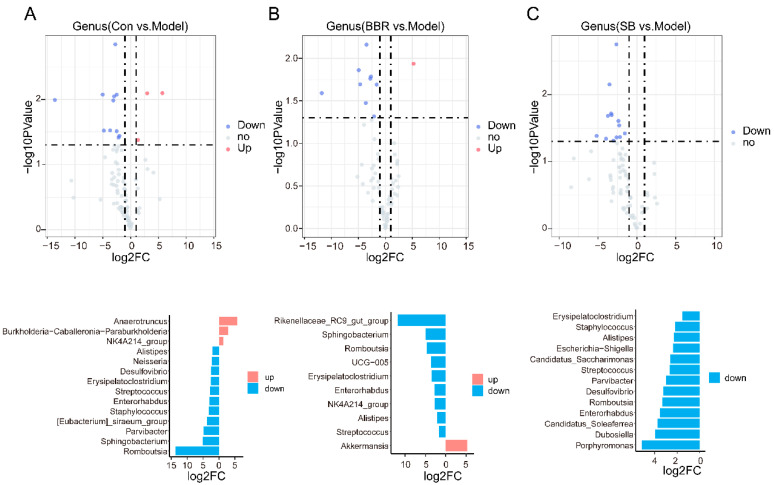
BBR and SB alter core species in UC mice under absolute quantification. (**A**) Up-regulated and down-regulated species in DSS groups compared to control group in absolute quantitative analysis. (**B**) Volcano map and two-way bar chart showing the core species altered by BBR in absolute quantitative analysis. (**C**) Volcano map and two-way bar chart showing the core species altered by SB in absolute quantitative analysis.

The volcano plot illustrates that the abundance of pathogenic and conditional pathogenic bacteria, such as Romboutsia, Sphingobacterium, Parvibacter, [Eubacterium]_siraeum_group, Staphylococcus, Enterorhabdus, Streptococcus, Erysipelatoclostridium, Desulfovibrio, Neisseria, and Alistipes, was significantly increased in the DSS group. Conversely, the abundance of beneficial bacteria such as NK4A214_group, Burkholderia-Caballeronia-Paraburkholderia, and Anaerotruncus were notably down-regulated (Figure 5A). Moreover, after BBR and SB supplementation, the abundance of Romboutsia, Enterorhabdus, Streptococcus, Erysipelatoclostridium, and Alistipes was significantly decreased. Specifically, BBR led to a significant decrease in Sphingobacterium, NK4A214_group, UCG-005, and Rikenellaceae_RC9_gut_group, while increasing Akkermansia levels. On the other hand, SB supplementation significantly reduced Porphyromonas, Dubosiella, Candidatus_Soleaferrea, Desulfovibrio, Parvibacter, Candidatus_Saccharimonas, Escherichia-Shigella, and Staphylococcus (Figure 5B,C).

The results indicate that BBR administration successfully restored the intestinal flora disorder induced by DSS, suppressed the growth of conditional pathogenic bacteria and pathogenic bacteria, and enhanced the growth of beneficial bacteria. In contrast, the administration of sodium butyrate further suppressed the growth of harmful bacteria but did not have a significant impact on beneficial bacteria.

### 3.4. Difference Between Absolute Quantification and Relative Quantification

The analysis results indicate that following DSS treatment, the alpha diversity of intestinal flora decreased and did not show improvement after supplementation with BBR and SB. Notably, the alpha diversity of the BBR group continued to decrease significantly in both methods, possibly due to the antibacterial properties of BBR [15,16]. Furthermore, beta diversity analysis using both quantitative methods revealed significant differences in community structure between the model group and the normal group. The community structure of the BBR group differed significantly from both the model and normal groups, whereas the SB group showed closer similarity to the normal group (Figure 2A–C and Figure 4A–C). These results suggest that there is no significant difference between absolute quantification and relative quantification methods in alpha and beta diversity analysis.

In absolute quantification, the total number of species in the model group was much higher than in the normal group, particularly in the Bacteroidata and Firmicutes phyla. Following BBR supplementation, the total number of species increased, especially the number of Verrucomicrobiota phyla, whereas the total number of species decreased significantly after SB supplementation, approaching levels seen in the normal group. However, in relative quantification, the amount of Bacteroidata in the model group was similar to that of the normal group; surprisingly, the SB group had the highest amounts of species in the Bacteroidata phylum among all groups, contrary to fact (Figure 2D,E and Figure 4D,E).

In both relative quantification and absolute quantification analysis, the abundances of *Sphingobacterium*, *Romboutsia*, and *Erysipelatoclostridium* in model groups were significantly up-regulated, while the abundances of *NK4A214_group*, *Anaerotruncus*, and *Burkholderia-Caballeronia-Paraburkholderia* were down-regulated. Post-administration of SB, only the abundance of *Enterorhabdus* was significantly down-regulated in both quantitative methods. Moreover, absolute quantification weakened the proliferation-promoting effect of SB on *Butyricimonas*, *Harryflintia*, and *Family_XIII_AD3011_group*, further emphasis its inhibitory effect on some pathogenic bacteria. Following BBR administration, only *Akkermansia* was significantly up-regulated in both quantitative analyses, which was consistent with extensive previous studies [17,18] (Figure 3A–C and Figure 5A–C). To further investigate whether relative quantitative sequencing might lead to spurious correlations between variables, a Spearman correlation analysis was conducted between inflammatory factors and key bacterial genera identified by the two analytical methods. As shown in Figure S2, the co-up-regulated *Akkermansia* genus in both analytical methods exhibited a more significant negative correlation with inflammatory factors in both colon and serum under absolute quantitative conditions.

### 3.5. Individual-Based Meta-Analysis of BBR-Regulated Gut Microbiota Across 13 Cohorts

To further elucidate the regulatory effects of BBR on intestinal flora and to distinguish between relative and absolute quantitative sequencing, the intestinal flora profile following BBR administration was analyzed utilizing the available 16S rRNA gene relative quantitative sequencing dataset, and these findings were subsequently compared with our sequencing results. A total of 264 relevant publications were retrieved from the PubMed database using ‘BBR’ and ‘gut microbiota’ as keywords. Among these, 10 articles provided raw sequencing data accessible via NCBI, identified by the following numbers: PRJNA982533 [19], PRJNA789741 [20], PRJNA848954 [21], PRJNA769853 [22], PRJNA723444 [23], CRA004451 [24], PRJNA753953 [25], PRJNA594263 [26], PRJNA681546 [27], and PRJNA667196 [28]. Additionally, three articles included the relative abundance of microorganisms directly in their Supplementary Materials [29,30,31].

To further elucidate the regulatory effect of BBR on gut microbiota as observed in previous studies, we first analyzed the key bacterial genera that were significantly up-regulated and down-regulated across 13 documents. In total, 15 genera were identified, including *Akkermansia*, *Lachnospiraceae_NK4A136_group*, *Bacteroides*, *Helicobacter*, *Odoribacter*, *Clostridia_UCG-014*, *Parabacteroides*, *Rikenellaceae_RC9_gut_group*, *Parasutterella*, *Alistipes*, *Erysipelatoclostridium*, *Blautia*, *Roseburia*, *NK4A214_group*, *Proteus*, and *UCG-005*. Notably, some studies indicate that BBR down-regulates the abundance of certain bacterial genera, such as *Bacteroides* and *Blautia*, while other studies have reported the opposite results (Supplementary Table S1).

**Figure 6:**
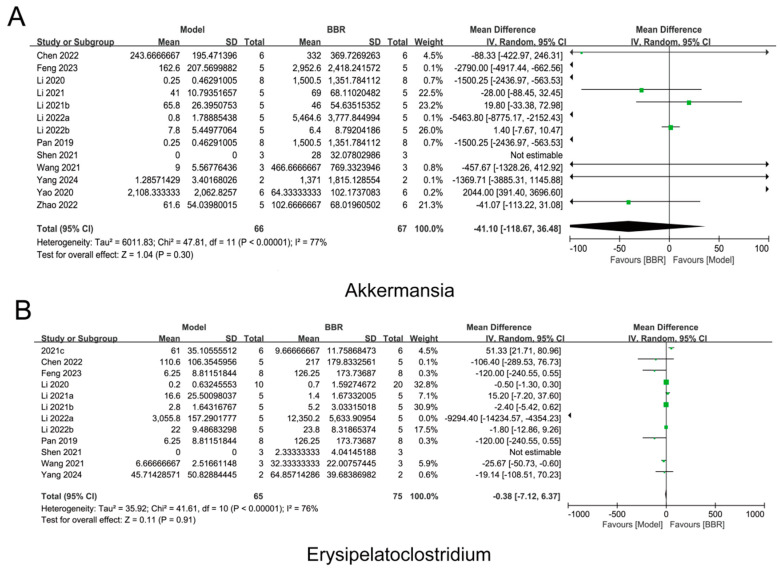
Gut bacteria consistent with absolute quantitative regulation [19,20,21,22,23,24,25,26,27,28,29,30,31]. (**A**,**B**) Forest plot illustrating a meta-analysis of bacteria regulated by BBR. (**A**) *Akkermansia* and (**B**) *Erysipelatoclostridium*. The diamond represents the 95% confidence interval (CI) of the combined effect size, with its midpoint indicating the point estimate and its length reflecting the range of the CI. The small squares denote the point estimates of the odds ratios (ORs) for each included study, and the horizontal lines associated with them represent the 95% CIs of the ORs. If a horizontal line crosses the null line, it indicates no statistically significant association between the study factor and the outcome. If the line lies to the right of the null line, it suggests a positive association between the study factor and the occurrence of the outcome event; conversely, if the line lies to the left, it implies a negative association.

**Figure 7:**
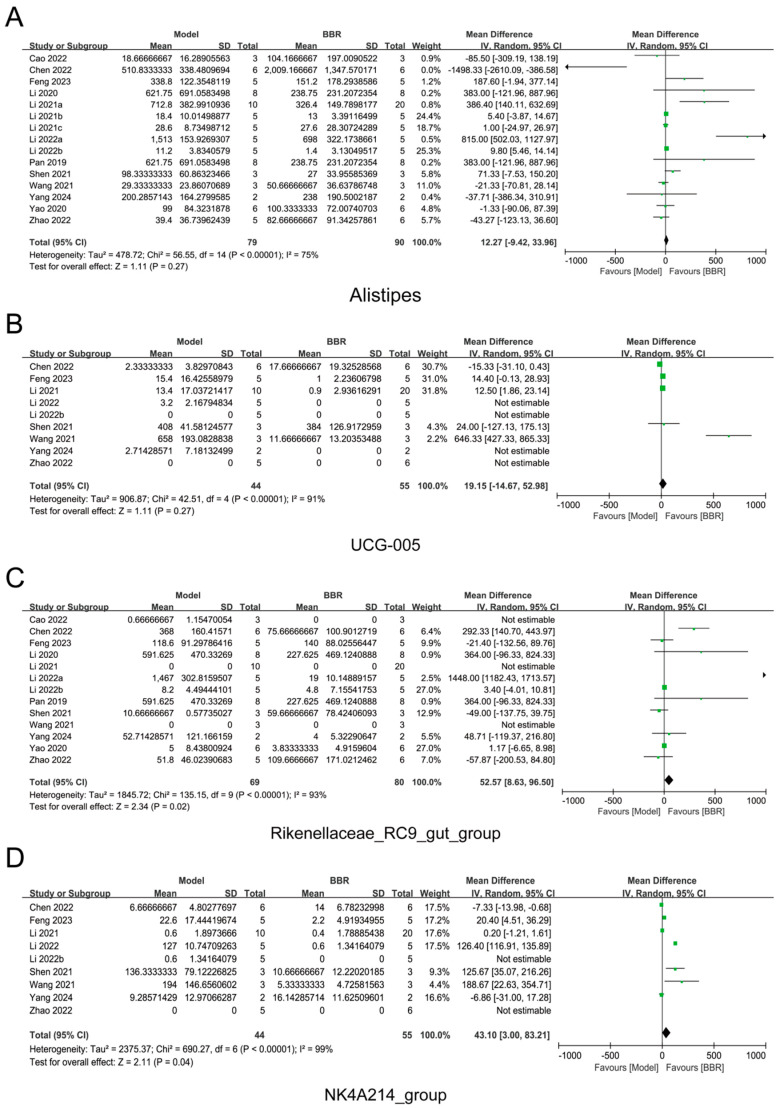
Gut bacteria contrary to absolute quantitative regulation [19,20,21,22,23,24,25,26,27,28,29,30,31]. (**A**–**D**) Forest plot illustrating a meta-analysis of bacteria regulated by BBR. (**A**) *Alistipes*. (**B**) *UCG-005*. (**C**) *Rikenellaceae_RC9_gut_group.* (**D**) *NK4A214_group*. The diamond represents the 95% confidence interval (CI) of the combined effect size, with its midpoint indicating the point estimate and its length reflecting the range of the CI. The small squares denote the point estimates of the odds ratios (ORs) for each included study, and the horizontal lines associated with them represent the 95% CIs of the ORs. If a horizontal line crosses the null line, it indicates no statistically significant association between the study factor and the outcome. If the line lies to the right of the null line, it suggests a positive association between the study factor and the occurrence of the outcome event; conversely, if the line lies to the left, it implies a negative association.

Further meta-analysis found that the abundance of *Akkermansia* increased while the abundance of *Erysipelatoclostridium* decreased following BBR administration, which was consistent with both our relative and absolute quantification results (Figure 6). Surprisingly, the meta-analysis results indicated that BBR up-regulated the expression of *Alistipes*, *UCG-005*, *Rikenellaceae_RC9_gut_group*, and *NK4A214_group*, which was in contrast to our relative and absolute sequencing results (Figure 7). This result suggests that previous relative quantitative analyses do not accurately reflect the regulatory effects of drugs on the gut microbiota and are prone to misleading the experimental conclusions, or even leading to completely opposite conclusions. Additionally, the meta-analysis revealed that the abundance of beneficial butyrate-producing bacteria, such as *Roseburia*, *Blautia*, and *Clostridia_UCG-014*, increased after BBR treatment (Figure S3), aligning with several previous reports [22,32,33].

## 4. Discussion

The gut microbiota has garnered growing attention as a therapeutic frontier for multiple diseases, yet prevailing assessment approaches for intestinal flora remain predominantly based on relative quantification. In this study, firstly we confirmed the beneficial effects of BBR and SB on colitis. We then compared the regulatory effects of BBR and SB on gut microbiota employing both relative and absolute quantitative methods. Finally, the regulatory effects of BBR on intestinal flora were systematically synthesized through a meta-analysis approach based on previous relative quantitative sequencing datasets, followed by comprehensive comparison with our original sequencing results. The results indicate that relative quantification solely captures the relative changes in microorganisms, failing to provide insights into the degree and direction of changes in species abundance. This limitation overlooks the overall shifts within the microbial community, potentially leading to the loss of critical information and the formulation of incorrect conclusions. In contrast, absolute quantification offers a more accurate representation of the actual changes within the sample’s bacterial community. This approach is vital for understanding the ecology of the microbiota and for effectively targeting interventions aimed at modulating the microbiota.

The human gut hosts a diverse array of microorganisms that play crucial roles in digestion, absorption, energy metabolism, immune regulation, and disease resistance [34,35]. Consequently, sequencing methods for gut microbiota are vital for studying these microorganisms and their related functions. However, relative quantitative methods overlook the absolute abundance of the entire microbial community, thereby failing to accurately characterize the interactions between microorganisms and between microorganisms and their hosts [36].

Our results indicate that after the administration of SB, the beta diversity observed through absolute quantitative sequencing was more aligned with the normal state compared to that observed through relative quantitative sequencing. This discrepancy may stem from the similarity in species composition between SB and Con, suggesting that the variations in analytical outcomes primarily reflect differences in the absolute abundance of the two species under investigation. Furthermore, significant differences in community composition were noted between the two analytical methods. This can be attributed to the fact that absolute quantification relies on the true absolute copy number of species, whereas relative quantification is based on standardized sequence numbers. The relative quantitative analysis adjusts the total microbial count across different samples to achieve consistency, which may obscure the actual differences in total microbial abundance among the samples. Therefore, to accurately assess the composition of microbiota and the interactions between species and their hosts, as well as to understand the potential implications of microbiota in pathology, physiology, and ecology, it is essential to employ quantitative analysis techniques that measure the absolute abundance of microbiota [37,38].

Increasing evidence suggests that gut microbiota significantly impacts human health; however, discrepancies exist regarding the specific drug-regulated bacteria across different studies. For instance, in the studies we included, the abundance of *Blautia* was significantly up-regulated following BBR administration in DHEA-induced PCOS rats, while it was significantly down-regulated in diabetic mice [29,39]. Similarly, the abundance of *Alistipes* was up-regulated in colorectal cancer mice after BBR administration but down-regulated in mice with metabolic syndrome [22,24]. These differences may be attributed to variations in pathological status, species, or gender. Therefore, comprehensive cross-cohort analysis is a more effective strategy for accurately understanding the regulatory effects of drugs on various microorganisms. Our research demonstrated the universal regulatory impact of BBR on different bacterial species through individual cross-queue gut microbiota analyses. Specifically, the analysis indicated that BBR up-regulated *Akkermansia muciniphila* while down-regulating *Erysipelatoclostridium*, findings that align with our absolute quantitative results and further validate the reliability of the absolute quantitative sequencing outcomes.

Although our findings demonstrate that absolute quantitative sequencing provides a more effective and realistic representation of the regulatory effects of drugs on gut microbiota compared to relative quantitative sequencing, this study has limitations requiring further validation. Due to current resource constraints, we did not incorporate metabolomic analysis. Metabolomics is critical for comprehensively elucidating the composition and dynamics of microbial community metabolites, offering key insights into biological system functionality and interactions. The absence of metabolomic data limits our ability to clarify relationships between murine fecal metabolites and gut microbiota, as well as internal correlations between representative metabolites and enriched genera following berberine intervention. Future studies should integrate metabolomic approaches when resources permit to further demonstrate the pivotal role of absolute quantitative analysis in microbiota research. Furthermore, given the inherent limitations of 16S rRNA sequencing, despite fluctuations in parameters such as false-positive rates, sensitivity to sparsity, and case/control balance, as well as spike-in recovery rates, relative quantification sequencing remains a core tool for analyzing the dynamics of microbial community structures [40,41,42]. Therefore, future microbiome research should rationally employ methods such as relative/absolute quantification sequencing and metabolic flux models to evaluate the regulatory effects of drugs on microbial communities. When comparing absolute and relative quantification, differences in normalization methods may introduce biases. Absolute quantification requires the addition of exogenous spike-in standards with known concentrations (e.g., synthetic oligonucleotides or reference strain DNA) combined with qPCR or flow cytometry to calibrate the total microbial load, converting sequencing reads into actual microbial absolute abundance (e.g., copies per gram). In contrast, relative quantification normalizes solely based on the total sequence count within samples (e.g., proportional normalization or centered log-ratio transformation, CLR), disregarding inter-sample variations in total microbial load. This methodological discrepancy may introduce biases. For instance, in low-biomass samples, absolute quantification can correct for spurious proportional shifts caused by fluctuations in DNA extraction efficiency, whereas relative quantification might amplify or obscure abundance differences of specific taxa due to variations in total microbial load. This is particularly critical in interventional studies (e.g., probiotic supplementation), where the impact of total microbial load changes on result interpretation must be explicitly discussed.

## 5. Conclusions

Our findings align with previous reports, demonstrating that BBR and SB can effectively ameliorate colitis. However, we observed significant differences in the β diversity and composition of the microbiota between relative quantitative and absolute quantitative sequencing methods. Furthermore, the comparison of our sequencing results with those from the meta-analysis reinforces the notion that relative quantitative sequencing is susceptible to misjudgments and false correlations. Overall, our study underscores the importance of absolute quantitative analysis in accurately representing the true microbial counts in a sample, elucidating real differences between groups, and evaluating the modulatory effects of drugs on the microbiome, which plays a vital role in the study of the microbiome.

## Figures and Tables

**Table 1:** The standards of DAI score.

Body Weight Loss	Stool Character	Bloody Feces
0	normal	no hematochezia
1–5%	slightly loose	no obvious hematochezia
6–10%	semi-formed dilute stool	darken feces
11–15%	mucoid stool	blood anus + darken feces
>15%	fluid stool	gross rectal bleeding

## Data Availability

All data are available from the corresponding authors upon reasonable request.

## Supplementary Materials

The following supporting information can be downloaded at: https://www.mdpi.com/article/10.3390/biom15030400/s1, Figure S1: Flowchart of the meta-analysis; Figure S2: Correlation analyses comparing relative and absolute quantitative analyses with inflammatory factors; Figure S3: BBR regulated Gut bacteria in meta-analysis; Table S1: Core flora regulated by BBR.
