# Supplementary material for: Absolute Quantitative Metagenomic Analysis Provides More Accurate Insights for the Anti-Colitis Effect of Berberine via Modulation of Gut Microbiota
Source: Biomolecules. 2025 Mar 11;15(3):400. doi: 10.3390/biom15030400 (PMC11940175; doi:10.3390/biom15030400)
Supplement: Supplementary file 1 [file biomolecules-15-00400-s001.zip › supplemental figure.pdf]

Supplementary Materials

Absolute quantitative metagenomic analysis provides more accurate insights for the anti-colitis effect of berberine via modulation of gut microbiota

Jiaguo Zhan[a], Jiale Cheng[a], Wenhui Chang[a], Yuying Su[a], Xixin Yue[a], Chongming Wu[a,b,c,*]

[a] School of Chinese Materia Medica, Tianjin University of Traditional Chinese Medicine, Tianjin 301617, China.

[b] State Key Laboratory of Chinese Medicine Modernization, Tianjin 301617, China.

[c] Tianjin Key Laboratory of Therapeutic Substance of Traditional Chinese Medicine, Tianjin 301617, China

[*] Corresponding authors: chomingwu@163.com (C.Wu)

Supplementary Figure S1

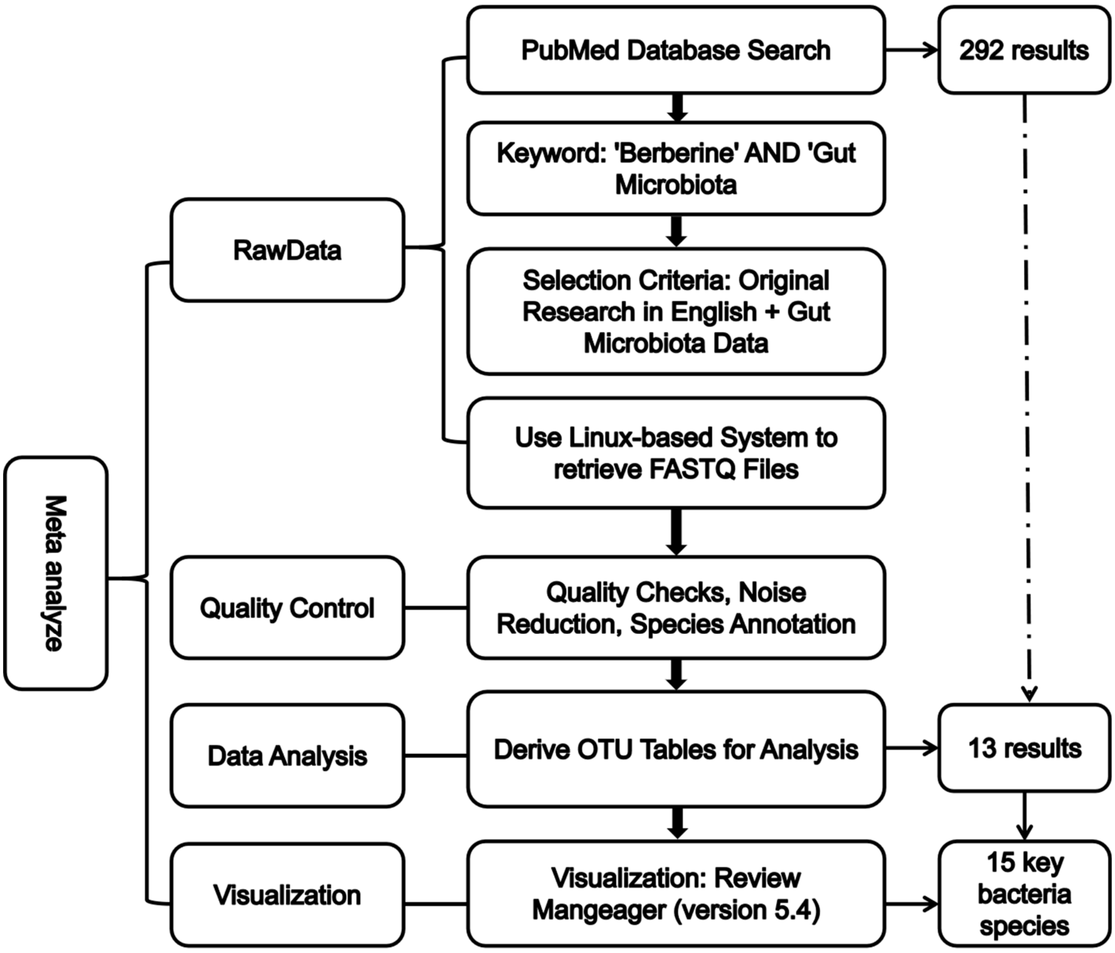

Figure S1. Flowchart of the meta-analysis.

Supplementary Figure S2

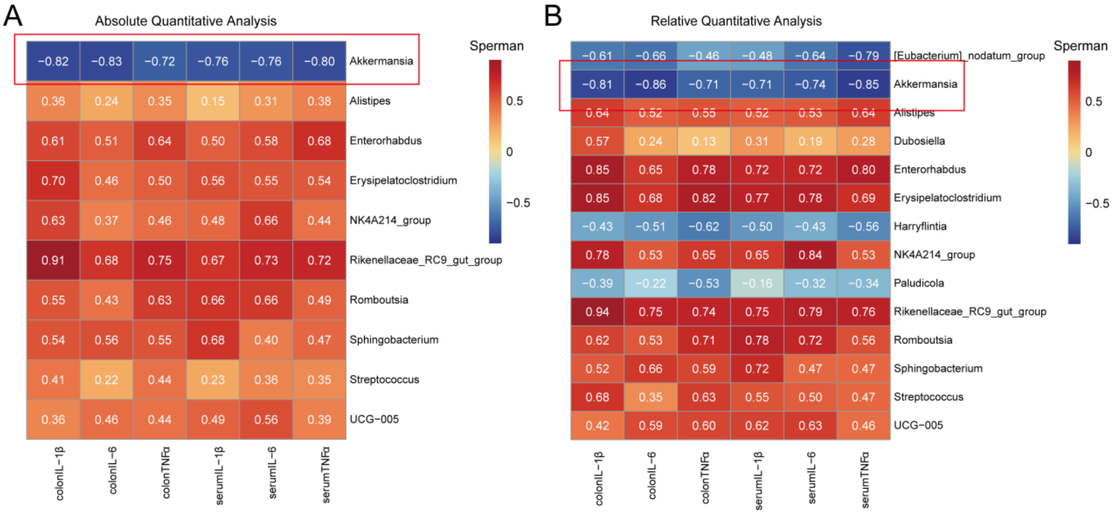

Figure S2. Correlation analyses comparing relative and absolute quantitative analyses with inflammatory factors.

Supplementary Figure S3

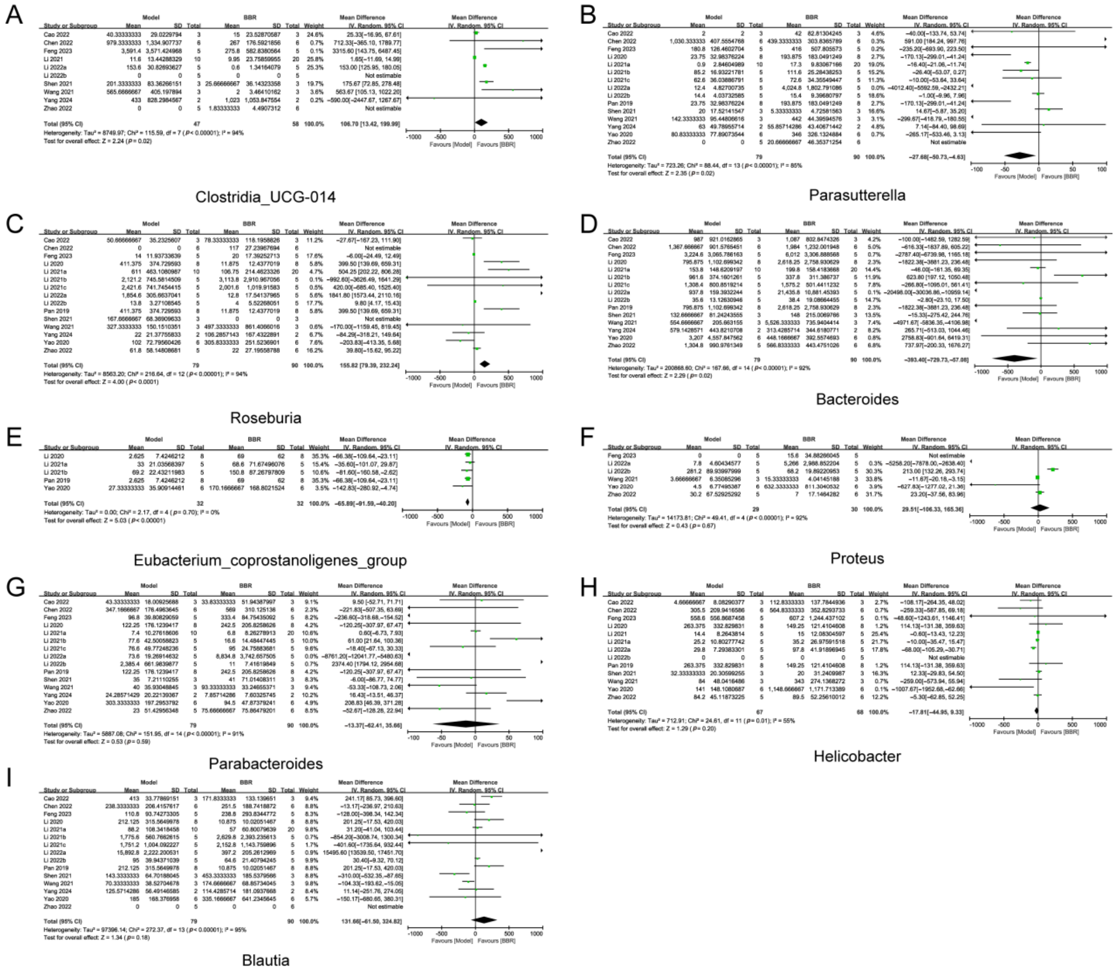

Figure S3. BBR regulated Gut bacteria in meta-analysis. (A-I) Forest plot illustrating a meta-analysis of bacteria regulated by BBR. (A) *Clostridia_UCG-014*. (B) *Parasutterella*. (C) *Roseburia*. (D) *Bacteroides*. (E) *Eubacterium_coprostanoligenes_group*. (F) *Proteus*. (G) *Parabacteroides*. (H) *Helicobacter* and (I) *Blautia*.
